# COVID-19 pandemic and families' utilization of well-child clinics and pediatric practices attendance in Germany

**DOI:** 10.1186/s13104-021-05562-3

**Published:** 2021-04-16

**Authors:** Mandy Vogel, Christoph Beger, Ruth Gausche, Anne Jurkutat, Roland Pfaeffle, Antje Körner, Christof Meigen, Tanja Poulain, Wieland Kiess

**Affiliations:** 1 Leipzig University Hospital for Children and Adolescents, LIFE Child, Leipzig University, Ph.-Rosenthal-Str. 27, 04103 Leipzig, Germany; 2 Department of Women and Child Health, Center for Pediatric Research, Leipzig University, Liebigstr. 20a, 04103 Leipzig, Germany; 3 Leipzig University Hospital for Children and Adolescents, CrescNet, Leipzig University, Liebigstr. 22a, 04103 Leipzig, Germany

**Keywords:** COVID-19, Health care utilization, Pediatrics

## Abstract

**Objective:**

The COVID-19 pandemic and the measures implemented to stop the pandemic had a broad impact on our daily lives. Besides work and social life, health care is affected on many levels. In particular, there is concern that attendance in health care programs will drop or hospital admissions will be delayed due to COVID-19-related anxieties, especially in children. Therefore, we compared the number of weekly visits to 78 German pediatric institutions between 2019 and 2020.

**Results:**

We found no significant differences during the first 10 weeks of the year. However, and importantly, from April, the weekly number of visits was more than 35% lower in 2020 than in 2019 (p = 0.005). In conclusion, the COVID-19 pandemic seems to relate to families´ utilization of outpatient well-child clinics and pediatric practice attendance in Germany.

**Supplementary Information:**

The online version contains supplementary material available at 10.1186/s13104-021-05562-3.

## Introduction

Measures implemented to stop the spread of COVID-19 infections may cause a pandemic of child neglect and abuse [1]. They have a broad impact on our daily routines, social life, economics, and wellbeing in societies around the world. In particular, health care is affected on many levels by the pandemic itself but also by the measures such as social distancing and quarantine for large numbers of people. There is concern that attendance to health care programs, urgent hospital admissions as well as necessary surgical or medical procedures are delayed, and this may affect young people in particular [2].

The German healthy baby and healthy child/youth outpatient clinics program has been in place for decades and is highly appreciated by families and society. Physicians and health insurances recognize its preventive aspects and the chances to discover impaired child development and acute and chronic disorders early. Attendance depends on the child’s age and the families' socioeconomic situation [3]. Early diagnostic tests such as vision and hearing tests included in the program enjoy a high level of acceptance among all social classes. With increasing age of the child, however, attendance rates decrease gradually. In addition, only 83% of the population with a lower social status attend (92% for high social status) [3]. We have hypothesized that the current COVID-19 pandemic would influence both general pediatric practice consultations and utilization of the German healthy baby and healthy child/youth clinics program.

## Main text

To answer this question, we have used data from our CrescNet database, a representative database of more than 430 pediatric practices in Germany. The CrescNet network was established in Germany in 1998 [4]. More than 130 pediatric practices and 21 pediatric endocrinology departments currently actively participate in data collection and networking [5]. Pediatricians record heights, weights, and growth velocities of all children in their care systematically and feed the data into the database at our center. Data are then continuously analyzed at the center regarding individual patients' growth (height and weight). Feedback to participating physicians is ensured via phone or E-mail. By October 2020, the data of more than 940,000 children and adolescents have been included, and more than 5 million data sets have been generated. The data source has already been used to answer important public health questions related to child health [5].

Using the data entry of the pediatric practices into the CrescNet system during the year 2020, we have asked whether or not the number of pediatric practice visits would decline or alternatively increase during the COVID-19 pandemic. Therefore, we compared the number of visits in March and April in 2020 to the number of visits in 2019, reflecting the changing patients´/families´ attitudes regarding pediatric healthcare utilization in Germany.

**Fig. 1:**
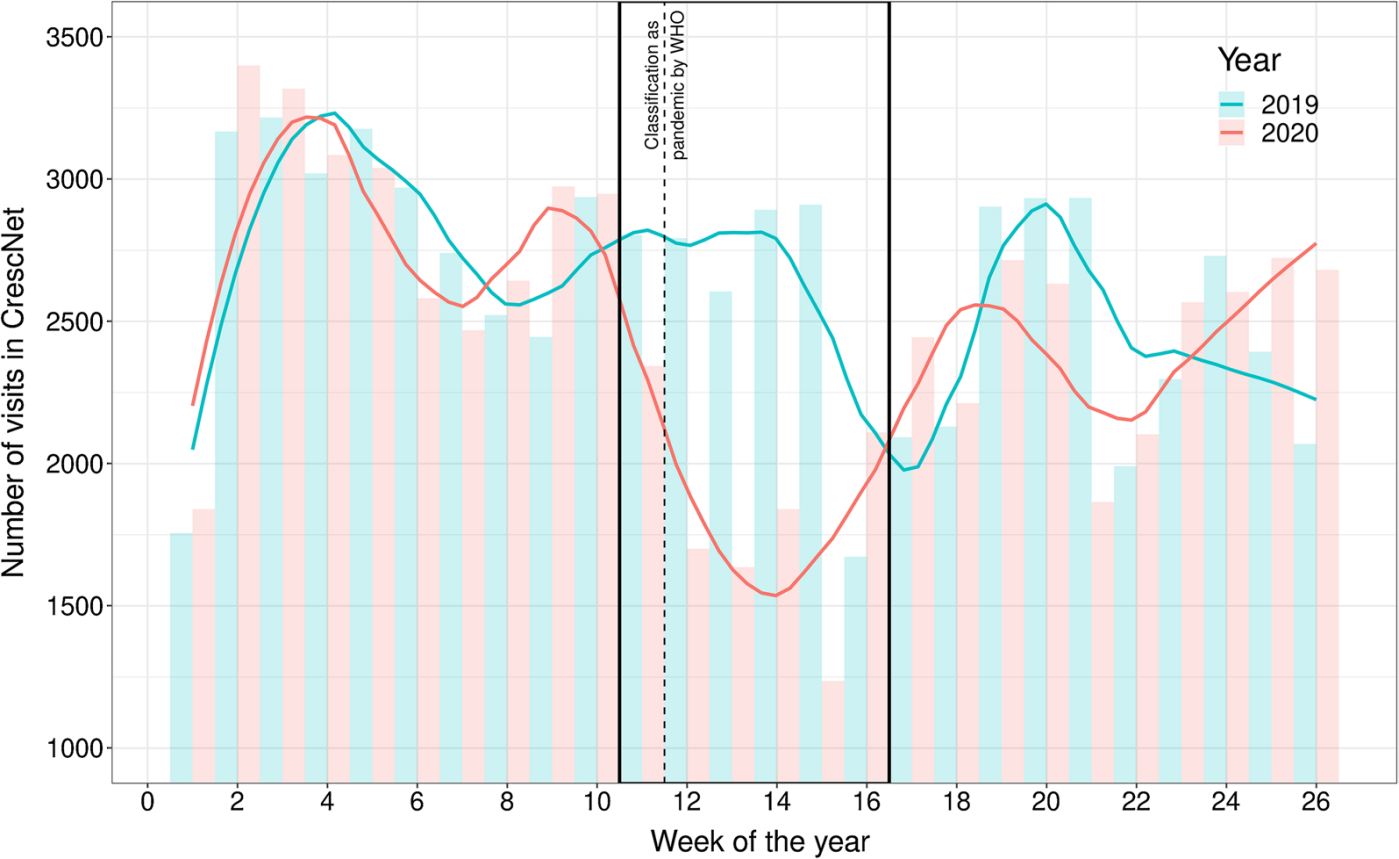
Weekly documented number of health visits in the CrescNet system from 78 German pediatric institutions transmitting electronically data on a regular basis during 2019 (blue) and 2020 (red). Since March 11, 2020, (calendar week 11) the spread of COVID-19 is classified as a pandemic by the WHO

Comparing the visit numbers in 2019 and 2020, a paired t-test revealed no significant change during the first 10 weeks of 2020 (diff = 35, p = 0.7). But afterward, starting in mid-March 2020 (week 10), 2 weeks before the lockdown was announced in Germany, documented visits to pediatric practices participating in the CrescNet system have been steadily decreasing with a then striking dip in numbers in April 2020 during the first wave of COVID-19 infections in Germany. The mean number of visits per week was significantly lower than during the same period the year before (diff = − 1050, p = 0.005, i.e., > 35%). Interestingly, there is also a continuously (non-significant) lower data entry during May and the first half of June, after the end of the lockdown, when very active communication and indeed public discussions on the dangers of the pandemic have steadily increased in Germany. With increasing numbers of adults being reported to carry COVID-19 and increasing numbers of COVID-19 patients in hospitals and even intensive care units, the number of visits to pediatric practices remained slightly lower than in 2019 (Fig. 1, Additional file 1). In contrast to the 2020 numbers, in previous years [5] exemplified for 2019, data entry numbers show only small undulations and fluctuations as are expected in relation to holiday seasons. There is no general telemedicine program in Germany. Visits were not substituted by online visits, well-child examinations require an in-person visit at the outpatient clinics.

Therefore, our results indicate a decline in well-child clinics, which is important because it supports the hypothesis of delayed diagnoses caused by COVID-19 or the related measures, as already reported for type I diabetes [6] or cancer [7].

In conclusion, the COVID-19 pandemic seems to relate to families´ utilization of well-child clinics and pediatric practice attendance in Germany. Whether or not this is due to families´ fears of their child or themselves becoming infected in medical facilities, or physicians being too busy to report and send in their data from their clinic to our study center remains uncertain. Alternatively, the hypothesis that people being simply overstressed or too busy to use routine services, such as well-child clinics, should be investigated. We hypothesize that people´s utilization of medical services change during a pandemic and this might lead to fundamental changes in health behaviors.

## Limitations

Our data do not contain information about the reasons for visits to pediatric practices (preventive medical checkup or visits because of acute or chronic diseases). Therefore, we cannot estimate the share in the decline caused by a decrease in infectious diseases also caused by measures against COVID-19. Moreover, whether or not other factors such as general societal factors, the distance between homes and the practices, and health issues might influence the attendance at child clinics cannot be examined.

## Supplementary Information

**Additional file 1.** Comparison of weekly visit numbers between the years 2019 and 2020.

## Data Availability

Data is included as Additional file 1.
